# Data on microbial and physicochemical assessment of mixed fruit wine produced from physically damaged fruits

**DOI:** 10.1016/j.dib.2018.05.104

**Published:** 2018-05-23

**Authors:** Deborah O. Oba, Oluwaseun J. Okunola, Solomon U. Oranusi, Hilary I. Okagbue

**Affiliations:** a Department of Biological Sciences, Covenant University, Ota, Nigeria; b Department of Mathematics, Covenant University, Ota, Nigeria

**Keywords:** Wine, Fermentation, Fruit, Microbial count, Replication, Statistics

## Abstract

The data described in this article was obtained in an experiment designed for the production of mixed fruit wine using physically damaged fruits in the process of fermentation. Three fruits (watermelon, pineapple and orange) were used in the wine production process. The fermentation process involved two stages; aerobic and anaerobic fermentation. The paper presents the data on microbial and physicochemical analyses carried out to monitor the fermentation and clarification processes.

**Specifications Table**

**Table:** 

Subject area	*Microbiology*
More specific subject area	*Industrial Microbiology*
Type of data	*Tables*
How data was acquired	*Microscope (Olympus, XSZ-107BN), colony counter (Stuart serial R000102178), spectrophotometer, Titration,pH meter (Hanna instruments.PH211 microprocessor) and weighing balance.*
Data format	*Raw, Analyzed.*
Experimental factors	Microbial counts, Physicochemical parameters measurement.
Experimental features	Three types of physically damaged fruits were used in the production of mixed fruit wine. During aerobic and anaerobic fermentations, changes in pH, Titratable Acidity (TTA), reducing sugar, alcohol content, specific gravity and total viable plate counts and total coliform count were monitored.
Data source location	*Ota, Ogun State, Nigeria.*
Data accessibility	*Data available within the article*

**Values of the data**

• The data presented here shows the microbial and physicochemical assessment of mixed fruit wine produced from physically damaged fruits.
• The data here could serve as a benchmark for other researchers that are willing to work on reducing post-harvest losses using damaged fruits.
• The data presented could give an understanding on how to channel waste to wealth.
• Multivariate statistical analysis can be applied for further exploration of the data.

## 1 Data

The data presented here represents the total viable plate counts and total coliform counts from the aerobic and anaerobic fermentation process of damaged fruit (watermelon, pineapple and orange) using pour plate method. Also, the measurements of the different physicochemical properties throughout the fermentation and clarification processes were presented. Fermentation ended on the 21th day of the experiment and clarification of the wine ran through six weeks. Analysis where carried out once every two weeks. During aerobic and anaerobic fermentations, changes in temperature, pH, titratable Acidity (TTA), specific gravity, alcohol content, reducing sugar and total viable plate and coliform counts were monitored and presented in Table 1, Table 2, Table 3, Table 4, Table 5, Table 6, Table 7.

**Table 1:** Changes in temperature (°C) during the fermentation process.

Days	Replicate 1	Replicate 2
0	–	33
1	28	29
2	30	29
3	29	29
4	27	28
5	28	29
6	28	28
7	28	28
14	27	29
21	27	28
28	27	28
42	27	27
56	27	27
63	27	27
64	27	27

**Remark:** Day 28- first week of clarification, Day 42-third week of clarification, Day 56-fifth week of clarification, Day 63-Sixth week of clarification, Day 64- Bottling of wine.

**Table 2:** Changes in pH during the fermentation process.

Days	Replicate 1	Replicate 2
0	5.73	5.72
1	4.93	4.95
2	4.64	4.63
3	4.55	4.55
4	4.59	4.49
5	4.50	4.50
6	4.49	4.49
7	4.39	4.40
14	4.25	4.27
21	4.10	4.12
28	4.05	4.07
42	4.05	4.03
56	3.96	3.95
63	3.93	3.94
64	3.93	3.93

**Remark:** Day 28- first week of clarification, Day 42-third week of clarification, Day 56-fifth week of clarification, Day 63-Sixth week of clarification, Day 64- Bottling of wine.

**Table 3:** Changes in titratable acidity (%) during the fermentation process.

Days	Replicate 1	Replicate 2
0	0.11	0.10
1	0.32	0.30
2	0.59	0.60
3	0.76	0.75
4	0.81	0.80
5	0.88	0.86
6	0.90	0.91
7	0.93	0.92
14	0.96	0.94
21	1.01	0.98
28	1.01	1.00
42	1.03	1.02
56	1.06	1.07
63	1.08	1.08
64	1.08	1.08

**Remark:** Day 28- first week of clarification, Day 42-third week of clarification, Day 56-fifth week of clarification, Day 63-Sixth week of clarification, Day 64- Bottling of wine.

**Table 4:** Changes in specific gravity during the fermentation process.

Days	Replicate 1	Replicate 2
0	1.0420	1.0421
1	1.0400	1.0401
2	1.0390	1.0391
3	1.0331	1.0332
4	1.0285	1.0283
5	1.0225	1.0222
6	1.0155	1.0153
7	1.0070	1.0072
14	1.0020	1.0021
21	0.9950	0.9950
28	0.9925	0.9927
42	0.9922	0.9920
56	0.9920	0.9920
63	0.9900	0.9910
64	0.9900	0.9900

**Remark:** Day 28- first week of clarification, Day 42-third week of clarification, Day 56-fifth week of clarification, Day 63-Sixth week of clarification, Day 64- Bottling of wine

**Table 5:** Changes in alcohol content (%) during the fermentation process.

Days	Replicate 1	Replicate 2
0	0	0
1	0.26	0.26
2	0.39	0.39
3	1.17	1.17
4	1.77	1.80
5	2.60	2.60
6	3.50	3.50
7	4.40	4.41
14	4.60	4.60
21	6.18	6.18
28	6.50	6.50
42	6.54	6.60
56	6.60	6.60
63	6.80	6.80
64	6.80	6.80

**Remark:** Day 28- first week of clarification, Day 42-third week of clarification, Day 56-fifth week of clarification, Day 63-Sixth week of clarification, Day 64- Bottling of wine.

**Table 6:** Changes in the sugar reduction (g/l) during the fermentation process.

**Days**	**Replicate 1**	**Replicate 2**
0	24.060	24.072
1	20.084	20.082
2	14.504	14.501
3	8.621	8.620
4	6.431	6.429
5	2.740	2.742
6	1.635	1.634
7	1.600	1.601
14	1.480	1.480
21	1.311	1.312
28	1.310	1.311
42	1.308	1.308
56	1.304	1.303
63	1.301	1.300
64	1.300	1.300

**Remark:** Day 28- first week of clarification, Day 42-third week of clarification, Day 56-fifth week of clarification, Day 63-Sixth week of clarification, Day 64- Bottling of wine.

**Table 7:** The microbial counts (cfu/ml) during the fermentation process.

Days	TVC	TCC
0	–	–
1	6.0×10^4^	0
2	8.5×10^4^	0
3	1.6×10^5^	0
4	1.95×10^5^	0
5	5.5×10^4^	0
6	2.5×10^4^	0
7	1.6×10^4^	0
14	4.0×10^3^	0
21	2.0×10^3^	0
63	1.0×10^3^	0
64	0	0

**Remark:** Day 28- first week of clarification, Day 42-third week of clarification, Day 56-fifth week of clarification, Day 63-Sixth week of clarification, Day 64- Bottling of wine, TVC- Total viable counts; TCC- Total coliform counts.

## 2 Experimental design, materials and methods

Highly acceptable wines can be made from practically all fruits. Wine can be fermented with yeast that occurs naturally in fruits and even damaged fruits. Details on the history of wine making using fruits, fermentation process, must preparation; the effect of yeast in wine production, aging, clarification, packaging/bottling, quality assessment and evaluation of wines of different fruits can be found in [1], [2], [3], [4], [5], [6], [7], [8], [9], [10], [11], [12], [13]. Related analysis can be explored, see [14], [15], [16], [17], [18] for details.

### 2.1 Must preparation

Physically damaged fruits were obtained from selected markets in Ota, Ogun State Nigeria. Different treatment measures were carried out on the fruits, which are; rinsing with sterile distilled water, hot water and chemical treatments. The fruits were weighed, washed, peeled, sliced, rewashed, seeds removed for the case of oranges and then reweighed. The fruits were blended with a sterile blender using counter top blender into puree, and then filtered and mixed with sterile distilled water (1:1 w/v).

### 2.2 Fermentation

Two fermentors were used in this experiment; the first is a primary fermentor which is for the aerobic fermentation and the secondary fermentation which is for the anaerobic fermentation.

In the primary fermentor, the mixed fruit wine were mixed with known amount of sugar and yeast nutrient, pectinase, potassium metabisulphite and the prepared starter culture were mixed and stirred every 12 hours with daily analysis of temperature, pH, specific gravity, alcohol content and reducing sugar. The primary fermentation lasted for 7 days.

The mixed fruit wine was then transferred to the secondary fermentor aseptically with physiochemical analysis on a weekly basis of temperature, pH, specific gravity, alcohol content and reducing sugar. The whole fermentation period lasted for 21 days and after which bentonite clay was added to aid clarification of the wine. This process lasted for six weeks. The microbial analysis was by standard microbiological methods, a 6 fold serial dilution was performed. Aliquot of the sample was inoculated into a Nutrient agar (NA) for total viable count (TVC) and MacConkey agar for coliform count using the pour plate method. Cultures were allowed to grow for 18–24 hours after which the resulting colonies were enumerated using a colony counter. Colony counts were converted to colony forming units using the formula below;

$$\mathrm{Colonyformingunit}=\frac{\mathrm{NoofColonies}}{\mathrm{volumeplated}}÷\mathrm{dilutionfactorcfu}/\mathrm{ml}$$

The microbial counts presented as the total viable count (TVC) and total coliform counts (TCC|) is shown in Table 7.

In the determination of titratable acidity 6grams of the sample was weighed into 100 ml beaker and 5oml of distilled water was added to the sample. This was titrated with 0.1 M NaOH solution to give a faint pink colour. 1 ml of 1% aqueous alcoholic phenolphthalein indicator solution was added. The calculation of the titratable acidity was done using the formula below;

$$\mathrm{Titratableacidity}(\%)\;=\frac{\mathrm{MlsofNaOHused}*0.1\mathrm{NNaOH}*\mathrm{multiequivalentfactor}(0.064)}{\mathrm{Gramsofsample}}\times 100$$

Specific gravity was determined by using a 25 ml specific gravity bottle which was cleaned with distilled water, dried in an oven at 50°C and allowed to cool in dessicator. The weight of the dry bottle was recorded as W_1_, The bottle was then filled with distilled water and the weight was recorded as W_2_. The bottle was emptied and filled with the wine sample and weight was recorded as W_3_. The specific gravity of the sample was calculated thus;

$$\mathrm{Speci}\mathrm{ficGravity}=\frac{W_{3}-W_{1}}{W_{2}-W_{1}}$$

The alcohol content was calculated using the data from the specific gravity;

Alcoholcontentbyvolume(%)=(Originalgravity−Finalgravity)*131.25

In the estimation of reducing sugar in wine samples, One ml of 3, 5-Dinitrosalicyclic acid (DNS) was added to 1 ml of supernatant of sample, in a test tube and the mixture heated in boiling water for 10 minutes. The test tube was cooled rapidly in tap water and the volume adjusted to 12 ml with distilled water. A blank containing 1 ml of distilled water and 1 ml of DNS was prepared. The optical density of the sample was read against the blank in the spectrophotometer or 540 nm absorbance. The concentration of reducing sugar in the supernatant was estimated from the glucose standard curve.

$$\mathrm{Reducingsugar}\;(g/L)=\;\frac{\mathrm{AbsorbanceofTest}}{\mathrm{Absorbanceofstandard}}\times 100$$

### 2.3 Statistical tests

Paired sample t- tests are conducted to determine the significant difference in the means of thee replicates. Null hypothesis implies that there is no significant mean difference and the alternative hypothesis implies otherwise. Small sample sizes necessitated the use of t-test. Three distinct tables are obtained for each parameter which is paired sample statistics, paired sample correlations and paired sample test. These are shown in Table 8, Table 9, Tablee 10, Table 11, Table 12, Table 13, Table 14, Table 15, Table 16, Table 17, Table 18, Table 19. Paired sample tests of changes in alcohol content (%) and sugar reduction were not considered because the values of the replicates are almost the same.

**Table 8:** Paired sample statistics of changes in temperature (°C).

		Mean	N	Std. Deviation	Std. Error Mean
	Replicate 1	25.8000	15	7.19325	1.85729
				
	Replicate 2	28.4000	15	1.50238	0.38791

**Table 9:** Paired sample correlation of changes in temperature (°C).

		N	Correlation	Significance
	Replicate 1 & Replicate 2	15	–0.798	0.000

**Tablee 10:** Paired samples test of changes in temperature (°C).

Statistic	Value
mean (paired difference)	–2.600000
Standard deviation (paired difference)	8.441395
t	–1.192902
Degrees of freedom	14
Significance (2 tailed)	0.252733

**Table 11:** Paired sample statistics of changes in pH.

		Mean	N	Std. Deviation	Std. Error Mean
	Replicate 1	4.4060	15	0.47634	0.12299
				
	Replicate 2	4.4027	15	0.47227	0.12194

**Table 12:** Paired sample correlation of changes in pH.

		N	Correlation	Significance
	Replicate 1& Replicate 2	15	0.998	0.000

**Table 13:** Paired sample test of changes in pH.

Statistic	Value
mean (paired difference)	0.003333
Standard deviation (paired difference)	0.029681
t	0.434959
Degrees of freedom	14
Significance (2 tailed)	0.670222

**Table 14:** Paired sample statistics of changes in titratable acidity (%).

		Mean	N	Std. Deviation	Std. Error Mean
	Replicate 1	0.8353	15	0.28760	0.07426
				
	Replicate 2	0.8273	15	0.28927	0.07469

**Table 15:** Paired sample correlation of changes in titratable acidity (%).

		N	Correlation	Significance
	Replicate 1 & Replicate 2	15	0.999	0.000

**Table 16:** Paired sample test of changes in titratable acidity (%).

Statistic	Value
mean (paired difference)	0.008000
Standard deviation (paired difference)	0.012071
t	2.566756
Degrees of freedom	14
Significance (2 tailed)	0.022378

**Table 17:** Paired sample statistics of changes in specific gravity.

		Mean	N	Std. Deviation	Std. Error Mean
	Replicate 1	1.0121	15	0.02037	0.00526
				
	Replicate 2	1.0122	15	0.02030	0.00524

**Table 18:** Paired Sample correlation of changes in specific gravity.

		N	Correlation	Significance
	Replicate 1 & Replicate 2	15	0.999	0.000

**Table 19:** Paired sample test of changes in specific gravity.

Statistic	Value
mean (paired difference)	–0.000067
Standard deviation (paired difference)	0.000302
t	–0.856145
Degrees of freedom	14
Significance (2 tailed)	0.406333
