# Supplementary material for: Data on microbial and physicochemical assessment of mixed fruit wine produced from physically damaged fruits
Source: Data Brief. 2018 May 23;19:678–86. doi: 10.1016/j.dib.2018.05.104 (PMC5997944; doi:10.1016/j.dib.2018.05.104)
Supplement: Supplementary file 1 — Supplementary material [file mmc1.pdf]

# COVENANT UNIVERSITY
## COLLEGE OF SCIENCE AND TECHNOLOGY
### DEPARTMENT OF MATHEMATICS

**CANAANLAND, KM 10, IDIROKO ROAD**
**P.M.B 1023, OTA, OGUN STATE, NIGERIA**
www.covenantuniversity.edu.ng, mat.covenantuniversity.edu.ng


**EXTERNAL MEMO**

**To:** Editor, Data in Brief
**From:** Corresponding Author
**Date:** 16th May, 2018
**Subject:** **Conflict of Interest**

I thereby declare the absence of any conflict of interest among the authors.

The authors have read the final draft and unanimously agreed that the paper be sent for review.


**Hilary I. Okagbue**
Department of Mathematics, College of Science and Technology, Covenant University, Ota.
Google Scholar: *Hilary Izuchukwu Okagbue*
Research Gate: *Hilary Okagbue*
Live DNA: 234.14453
Scopus ID: 56438006100
ORCID: 0000-0002-3779-9763
